# Screening and identification of lactic acid bacteria with α-glucosidase inhibiting activity

**DOI:** 10.3389/fmicb.2025.1579393

**Published:** 2025-07-23

**Authors:** Yi Liu, Zixian Yang, Ang Liu, Boyu Dong, Qiuping Yao, Xinglin Ran, Dequan Zhu

**Affiliations:** ^1^School of Chinese Ethnic Medicine, Guizhou Minzu University, Guiyang, Guizhou, China; ^2^Key Laboratory of Development and Utilization of Medical Resources of Ethnic Minorities in Guizhou Province, Guizhou Minzu University, Guiyang, China

**Keywords:** lactic acid bacteria, α-glucosidase, hypoglycemic, tolerance, probiotics

## Abstract

In this study, lactic acid bacteria were isolated from traditional fermented foods in Guizhou. The fermentation supernatant and cell disruption extract were used to screen lactic acid bacteria with α-glucosidase inhibitory activity, and the active lactic acid bacteria strains with potential hypoglycemic effect were screened. The results showed that 10 strains were screened from 27 strains of lactic acid bacteria with α-glucosidase inhibitory activity, of which 5 strains had good acid and bile salt tolerance. The best acid resistance was XG01, and the survival rate was as high as 98.5%. ST01 had the strongest tolerance to bile salt, up to 77.3%. The lactic acid bacteria cell extracts of HLG01, YR01, ST01 and XG01 could better inhibit the activity of α-glucosidase, and the inhibition rates were 39.27, 38.6, 38.53, and 34.4%, respectively. The fermentation supernatant of JR01 could better inhibit the activity of α-glucosidase, and the inhibition rate reached 33.8%. Through molecular biological identification, HLG01, YR01, and ST01 were were *Lactiplantibacillus plantarum*, XG01 was *Pediococcus pentosaceus*, and JR01 was *Weissella cibaria*. *Weissella cibaria* JR01 is the first reported lactic acid bacterium with α-glucosidase inhibitory activity. Five strains of lactic acid bacteria with good tolerance were screened out, which provided a strain basis for the subsequent study of the hypoglycemic effect of lactic acid bacteria and the development of various functional foods.

## 1 Introduction

Diabetes has become one of the major challenges in global public health, with its high incidence and severe complications posing a serious threat to human health. Type 2 diabetes mellitus (T2DM), a common chronic disease, is characterized by insulin resistance and the decline of pancreatic β-cell function, and patients typically present with abnormally elevated blood glucose levels (Sousa et al., 2020). Clinically, the main drugs used for diabetes treatment include acarbose, voglibose, and miglitol. Among these, acarbose, an α-glucosidase inhibitor, is a commonly used oral hypoglycemic drug that controls postprandial blood glucose by delaying carbohydrate decomposition and absorption (Wang et al., 2022). However, acarbose and other such drugs have certain limitations in clinical application. The primary side effects arise from increased undigested carbohydrates in the intestine, which can cause abdominal distension, diarrhea, and other gastrointestinal discomforts (Zeng et al., 2020). Some patients may also experience secondary failure, affecting long-term efficacy. In light of this, researchers are committed to exploring natural products in the hope of discovering potential substances from natural components that can effectively alleviate the side effects associated with the aforementioned synthetic drugs. This could provide a safer and more effective complementary solution for diabetes treatment.

In recent years, lactic acid bacteria (LAB) have been widely studied for their probiotic properties and significant role in fermented foods. Some LAB strains can inhibit α-glucosidase activity (Wang et al., 2022), showing potential in developing novel anti-diabetic functional foods. However, research on LAB from Guizhou’s fermented foods remains limited. The unique traditional processing and rich microbial diversity of fermented foods in Guizhou offer great potential for discovering new functional LAB.

Probiotics are beneficial microorganisms that, upon ingestion, can modify the composition of bacterial communities within the human small intestine, thus further exerting positive effects on human health (Liew et al., 2022). Probiotics play a role through various mechanisms, such as competing with pathogens for nutrients or adhesion sites, breaking down toxins, generating antimicrobial substances, and activating innate as well as adaptive immune systems (Yoon et al., 2022).

Lactic acid bacteria are ubiquitous and constitute a significant component of probiotics. As a food-grade microorganism recognized globally for its safety and edibility, Lactic acid bacteria have been extensively applied in the production of fermented foods and food preservation (Rudzki et al., 2021). Certain probiotic strains of Lactic acid bacteria exhibit a remarkable inhibitory impact on the enzyme activity of α-glucosidase present in the small intestine. This action can diminish the human body’s capacity to digest and absorb carbohydrates, thereby significantly curbing the postprandial blood glucose surge (Zhao et al., 2019).

Lactic acid bacteria play a pivotal role in maintaining human health. When selecting Lactic acid bacteria that are beneficial to the human body, the foremost consideration is their ability to survive in human gastric and intestinal juices after entering the body, enabling effective colonization within the human system (Hernández-Alcántara et al., 2018). Only when they can exist in the gastric and intestinal environments for a certain duration without being digested can they function optimally. Hence, screening Lactic acid bacteria with superior gastrointestinal fluid tolerance is a crucial aspect of identifying probiotic Lactic acid bacteria (Kusada et al., 2021).

α-glucosidase, also termed glucosyltransferase. It can catalyze the breakdown of maltose and sucrose in the intestinal tract following meals, which in turn leads to an enhancement in the rate of sugar absorption within the human body (Barber et al., 2021). Through the inhibition of α-glucosidase activity, the generation and uptake of glucose can be decelerated. This action contributes to the regulation of insulin levels in the body and reduces the postprandial blood glucose peak. Ultimately, it assists in normalizing blood glucose levels within the human body (Chávez-Silva et al., 2018).

Recent research into hypoglycemic effects has revealed that a variety of probiotic Lactic acid bacteria exhibit significant potential in the prevention or mitigation of diabetes. Evidence also indicates that probiotic Lactic acid bacteria can regulate the breakdown and synthesis of glucose-related compounds in human glucose metabolism (Jeong et al., 2021), thereby maintaining human blood glucose level stability. Lactic acid bacteria primarily control blood glucose levels by modulating the activity of intestinal α-glucosidase. The relationship between probiotic Lactic acid bacteria and hypoglycemia is gaining increasing attention from researchers both domestically and internationally. The development of Lactic acid bacteria-related probiotic foods also holds great promise for practical applications (Pinto et al., 2020). This study focused on lactic acid bacteria from fermented food in Guizhou, aiming to explore its inhibitory effect on α-glucosidase activity. This study not only fills the gap in the research of lactic acid bacteria from fermented food in Guizhou, but also provides new candidate strains for the development of new hypoglycemic functional foods. Screening of probiotic lactic acid bacteria with inhibition of α-glucosidase activity lays the foundation for the development of functional foods with hypoglycemic activity.

## 2 Materials and methods

### 2.1 Materials

Raw materials: Fermented foods (kimchi, sour soup, sauerkraut, fermented soybean paste, rice wine) and fruits (watermelon, dragon fruit) were all purchased from the market.

Experimental strains: Twenty-seven strains of lactic acid bacteria were isolated and purified from traditional fermented foods and fruits (kimchi, sour soup, sauerkraut, fermented soybean paste, rice wine, watermelon, dragon fruit) from Guizhou Province and used as experimental strains.

The MRS agar and broth media were sourced from Shanghai Bo Microbiology Technology Co., Ltd. The α-glucosidase, PNPG (p-nitrophenyl-α-d-glucopyranoside), and porcine bile salts were obtained from Beijing Solepol Science and Technology Co. PNP (p-nitrophenol) was acquired from Shanghai McLean Biochemical Technology Co., Ltd.

### 2.2 Methods

#### 2.2.1 Isolation and purification of strains

In this study, 100 μL of fermented food and fruit juices were added to 10 mL of sterile normal saline, mixed by oscillation, and serially diluted 10-fold with sterile normal saline. Then, 100 μL of diluted samples from three appropriate dilutions were spread-plated on MRS agar medium and incubated at 37°C for 24–48 h. Single colonies with different morphologies were picked, streaked on plates until uniform colonies were obtained. Colony characteristics were observed, and Gram staining was performed for preliminary lactobacilli identification. The identified strains from different sources were inoculated into sterile MRS broth. After incubation at 37°C for 24 h, repeated streaking was done to ensure pure cultures.

#### 2.2.2 Preparation of *Lactobacillus* fermentation supernatants and cell extracts

The preparation of lactic acid bacteria fermentation supernatant was based on the method described by Wang and Li (2022) with modifications. The culture broth was centrifuged at 4,000 rpm for 10 min to separate the supernatant from the bacterial cells. The supernatant was then filtered through a 0.22 μm membrane filter to obtain the fermentation supernatant for experimentation. For the preparation of cell extracts, the sediment was rinsed 2 ~ 3 times with 0.85% normal saline and resuspended. The cells were disrupted using an ultrasonic cell crusher at 45% power, operating for 10 s with a 5 s interval, for a total effective working time of 15 min. After disruption, the mixture was centrifuged at 12,000 rpm for 20 min in a high-speed refrigerated centrifuge at 4°C. The supernatant was collected and filtered through a 0.22 μm membrane filter to obtain the cell extract for experimentation.

#### 2.2.3 Determination of the inhibition rate of lactic acid bacteria on α-glucosidase activity

Following the method of Wang X. et al. (2021) and Wang Y. et al. (2021), first add the fermentation supernatant or cell extract of the Lactic acid bacteria to be tested and PNPG (p-Nitrophenyl-α-d-glucopyranoside) to normal saline for reaction. After a certain time, add the α-glucosidase solution. After the reaction is terminated, measure the absorbance value, which correlates with the free PNP amount. By comparing the sample and blank control inhibitory activities, lactic acid bacteria strains with strong inhibitory activity were screened.

#### 2.2.4 Determination of calcium dissolution circle of lactic acid bacteria

Prepare MRS solid medium with 0.5% calcium carbonate: Add 0.5 g of calcium carbonate to each 100 mL MRS agar medium, sterilize, cool slightly, and pour into the plate. After solidification, inoculate the cells and culture at 37°C for 48 h to observe the calcium-dissolving effect.

#### 2.2.5 Determination of acid resistance of lactic acid bacteria

Referring to the method of Kim et al. (2021) with slight modifications, the activated bacterial suspension was inoculated into MRS broth at pH 2.5 at a 1% inoculation volume and incubated at 37°C. Viable plate counts were performed at 0 and 3 h to calculate the survival rate. Three parallel trials were conducted, and the average was calculated to screen for strains with better acid resistance.

#### 2.2.6 Determination of bile salt tolerance of lactic acid bacteria

Referring to the method of Kim et al. (2021) with slight modifications, the activated bacterial culture was inoculated into MRS liquid medium containing 0.3% porcine bile salts at a 1% inoculation rate and incubated at 37°C. Viable plate counts were performed at 0 and 3 h, and three parallel trials were conducted. The survival rate was calculated to screen for strains with good bile salt tolerance.

#### 2.2.7 Morphological observation of strains

The Lactic acid bacteria, which showed inhibitory effects on α-glucosidase and had good probiotic characteristics, were isolated and inoculated onto MRS agar plates using the three-zone demarcation line method. They were then cultivated in a 37°C constant-temperature incubator for 48 h to examine the colonial morphology of the Lactic acid bacteria.

#### 2.2.8 Identification of the 16S rDNA gene of lactic acid bacteria

DNA extraction was performed as follows: (1) centrifuge 0.1 ~ 0.5 mL of bacterial culture at 12,0000 rpm (~13,400 × g) for 10 min and remove the supernatant completely; (2) resuspend the bacterial pellet in 200 μL of buffer GA by vortexing; (3) add 20 μL of Proteinase K solution and mix well; (4) add 220 μL of buffer GB, vortex for 15 s, and incubate at 70°C for 10 min. The solution should become clear. Briefly centrifuge to remove water droplets from the tube lid; (5) add 220 μL of anhydrous ethanol and mix well for 15 s. Briefly centrifuge again to remove water droplets from the tube lid; (6) transfer the resulting solution and any flocs to a CB3 adsorption column (placed in a collection tube) and centrifuge at 12,000 rpm (~13,400 × g) for 30 s. Discard the filtrate and place the CB3 column back into the collection tube; (7) add 500 μL of buffer GD (check if anhydrous ethanol has been added) to the CB3 column and centrifuge at 12,000 rpm (~13,400 × g) for 30 s. Discard the filtrate; (8) add 600 μL of wash buffer PW (check if anhydrous ethanol has been added) to the CB3 column and centrifuge at 12,000 rpm (~13,400 × g) for 30 s. Discard the filtrate; (9) repeat step 8; (10) place the CB3 column in the collection tube and centrifuge at 12,000 rpm (~13,400 × g) for 2 min. Discard the filtrate. Air-dry the CB3 column at room temperature for a few minutes to remove residual wash buffer; (11) transfer the CB3 column to a clean centrifuge tube. Add 50 ~ 200 μL of elution buffer TE droplet-by-droplet to the center of the adsorption membrane. Incubate at room temperature for 2–5 min and centrifuge at 12,000 rpm (~13,400 × g) for 2 min to collect the eluate in the centrifuge tube.

PCR amplification was performed using the primer set 27F (5-AGAGTTTGATCCTGGCTCAG-3) and 1492R (5-GGTTACCTTGTTACGACTT-3).

The 25 μL PCR reaction mixture contained the following components: 2.0 μL of genomic DNA, 1.25 μL of forward primer (10 μM), 1.25 μL of reverse primer (10 μM), 12.5 μL of Q5 High-Fidelity 2X Master Mix, and 8.0 μL of nuclease-free water. The mixture was gently flicked to mix, followed by a brief centrifugation to collect droplets from the tube walls. PCR amplification was carried out in a 0.2 mL centrifuge tube under the following conditions: initial denaturation at 98°C for 30 s, followed by 30 cycles of denaturation at 98°C for 10 s, annealing at 58°C for 30 s, and extension at 72°C for 45 s, with a final extension at 72°C for 5 min. After amplification, 3 μL of the PCR product was subjected to 1% agarose gel electrophoresis to verify the amplified fragment.

The PCR product gel recovery: PCR products were recovered with AxyPrep DNA gel recovery kit, the specific operation according to the kit instructions, the steps are as follows: (1) the agarose gel containing the target DNA was cut under the ultraviolet lamp and put into a clean centrifuge tube, and the weight was weighed. (2) buffer DE-A with three gel volumes was added and mixed evenly, and then heated at 75°C until the gel block completely melted; (3) add 0.5 Buffer DE-A volume of Buffer DE-B, mixed evenly; when the isolated DNA fragment was less than 400 bp, 1 gel volume of isopropanol was added. (4) The mixture was transferred to the DNA preparation tube 12,000 × g and centrifuged for 1 min. Waste filtrate; (5) the preparation tube was placed back into 2 mL centrifuge tube, 500 μL Buffer W1 was added, 12,000 × g was centrifuged for 30 s, and the filtrate was discarded. (6) Place the prepared tube back into 2 mL centrifuge tube, add 700 μL Buffer W2,12,000 × g, centrifuge for 30 s, and discard the filtrate. Centrifugation with 700 μL Buffer W2, 12,000 × g for 1 min; (7) the preparation tube was placed back into a 2 mL centrifuge tube, centrifuged at 12,000 × g for 1 min; (8) place the preparation tube in a clean 1.5 mL centrifuge tube, add 25–30 μL deionized water to the center of the preparation membrane, and stand at room temperature for 1 min. Centrifuge 12,000 × g for 1 min to elute DNA.

Sequencing and analysis: the purified PCR products of each strain were sequenced by ABI3730-XL.

Sequence analysis: NCBI Blast program was used to compare the assembled sequence files with the data in the NCBI database, and the species information with the greatest similarity to the species to be tested was obtained, which was the identification result.

#### 2.2.9 Data analysis

IBM SPSS Statistics 24 software was used to analyze the significance of the data, and the significance level was set to (*p* < 0.05). The graphics were drawn using Orgin 2024 software. The results were expressed as the mean ± standard deviation of at least three data, and there were significant differences between different numbers with the same parameters. The sequencing results of lactic acid bacteria strains were analyzed by BLAST (NCBI) software and MEGA-X software to construct phylogenetic tree.

## 3 Results and analysis

### 3.1 Inhibitory activity of lactic acid bacteria on α-glucosidase

#### 3.1.1 Inhibitory activity of lactic acid bacteria fermentation supernatant on α-glucosidase

**Figure 1:**
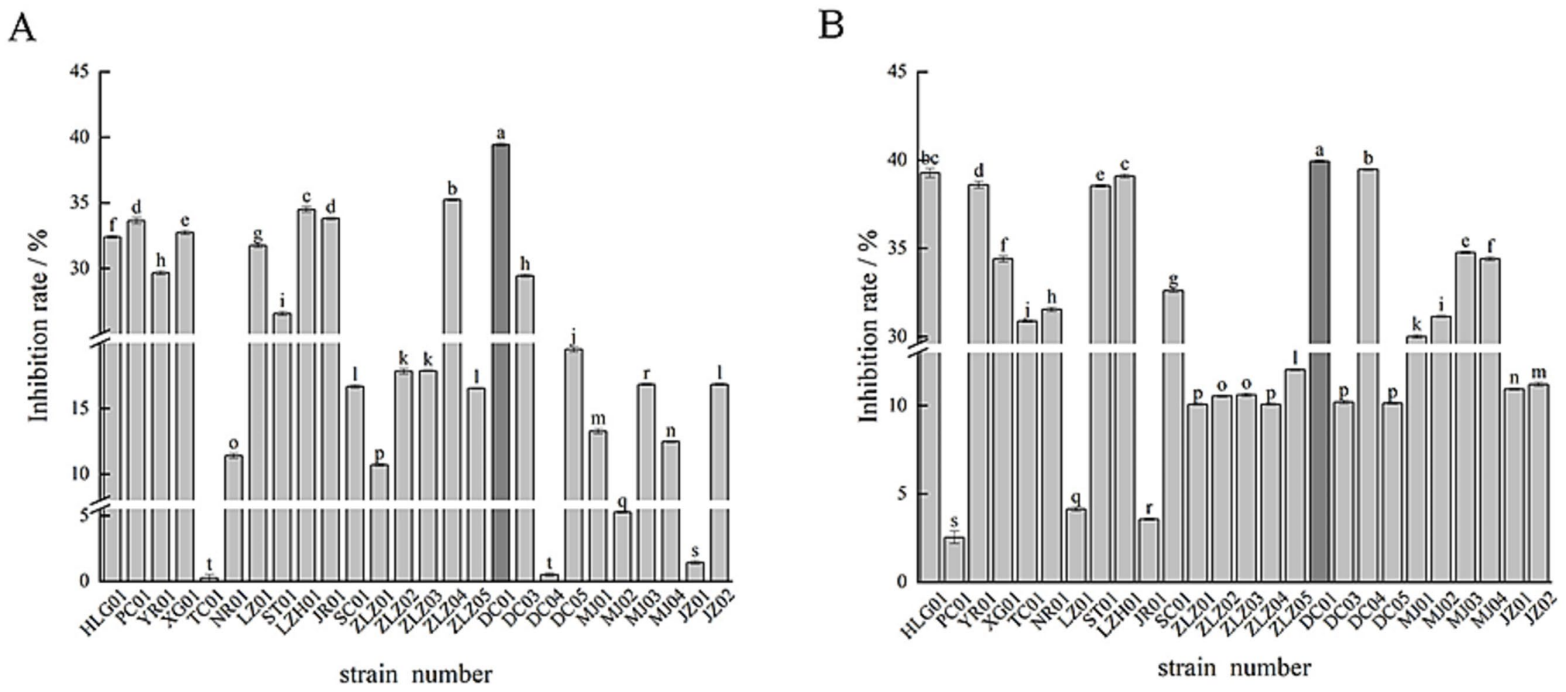
Inhibitory effect of lactic acid bacteria fermentation supernatant on α-glucosidase **(A)**, inhibitory effect of lactic acid bacteria cell extract on α-glucosidase **(B)**.

The inhibitory effect of various Lactic acid bacteria fermentation supernatants on α-glucosidase is illustrated in Figure 1A. As can be seen from Figure 1A, there are differences in the inhibitory effects of different strains on α-glucosidase in the experiment. Among them, the fermentation supernatant of DC01 showed the most significant inhibitory effect on α-glucosidase activity, reaching 39.4%. The strain MBEL 1397 proposed by Kwun et al. (2020) exhibited inhibitory activity of 3.91% ± 0.25%, while TC01 had the lowest inhibition rate of only 0.27%. In total, 12 strains had an inhibition rate of over 20%, 10 strains had an inhibition rate between 10 and 20%, and 5 strains had an inhibition rate of less than 10%.

#### 3.1.2 Inhibitory activity of lactic acid bacteria cell extract on α-glucosidase

The α-glucosidase inhibitory activity of lactic acid bacteria cell extracts is shown in Figure 1B. As depicted in Figure 1B, the cell disruption extracts of 15 strains out of the 27 tested exhibit markedly diverse inhibitory effects on α-glucosidase. Notably, DC01 demonstrated the highest inhibitory efficacy with an inhibition rate as high as 39.93%. Conversely, PC01 displayed the lowest inhibition rate at merely 2.53%. Among these strains, 15 have an inhibition rate exceeding 30%, while the remaining 12 strains have an inhibition rate below 12%.

### 3.2 Physiological characteristics of lactic acid bacteria

#### 3.2.1 Acid production performance of lactic acid bacteria

**Figure 2:**
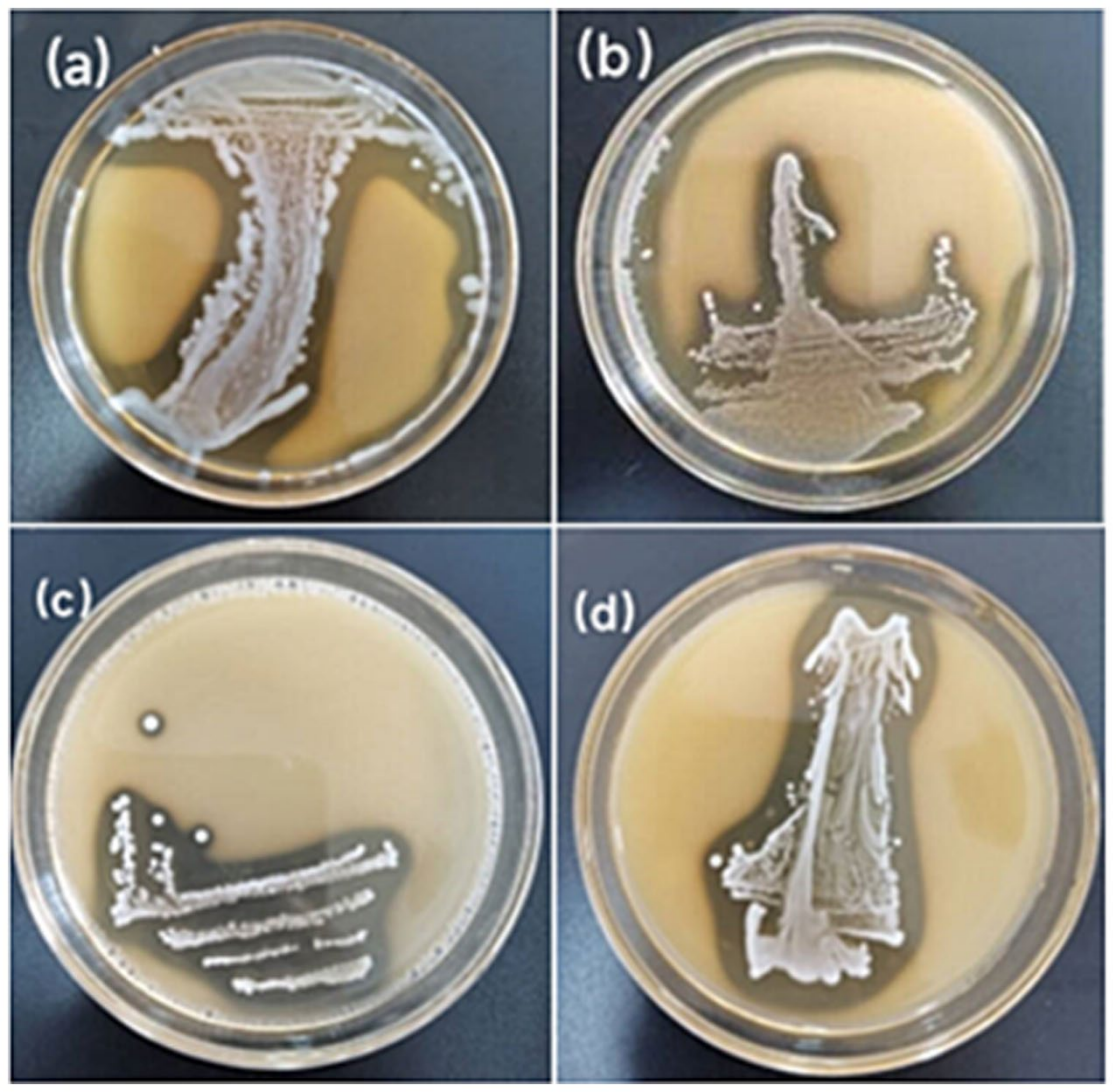
Assessment of calcium solubilization halos by lactic acid bacteria. **(a)** HLG01; **(b)** YR01; **(c)** XG01; **(d)** ST01.

The 10 Lactic acid bacteria strains with α-glucosidase inhibitory activity exhibited a pronounced calcium-dissolving circle effect in CaCO_3_ MRS solid medium, suggesting these strains can generate CaCO_3_-dissolving acidic substances. Colony morphologies of some strains are partially shown in Figure 2.

#### 3.2.2 Acid and bile salt tolerance of lactic acid bacteria

**Figure 3:**
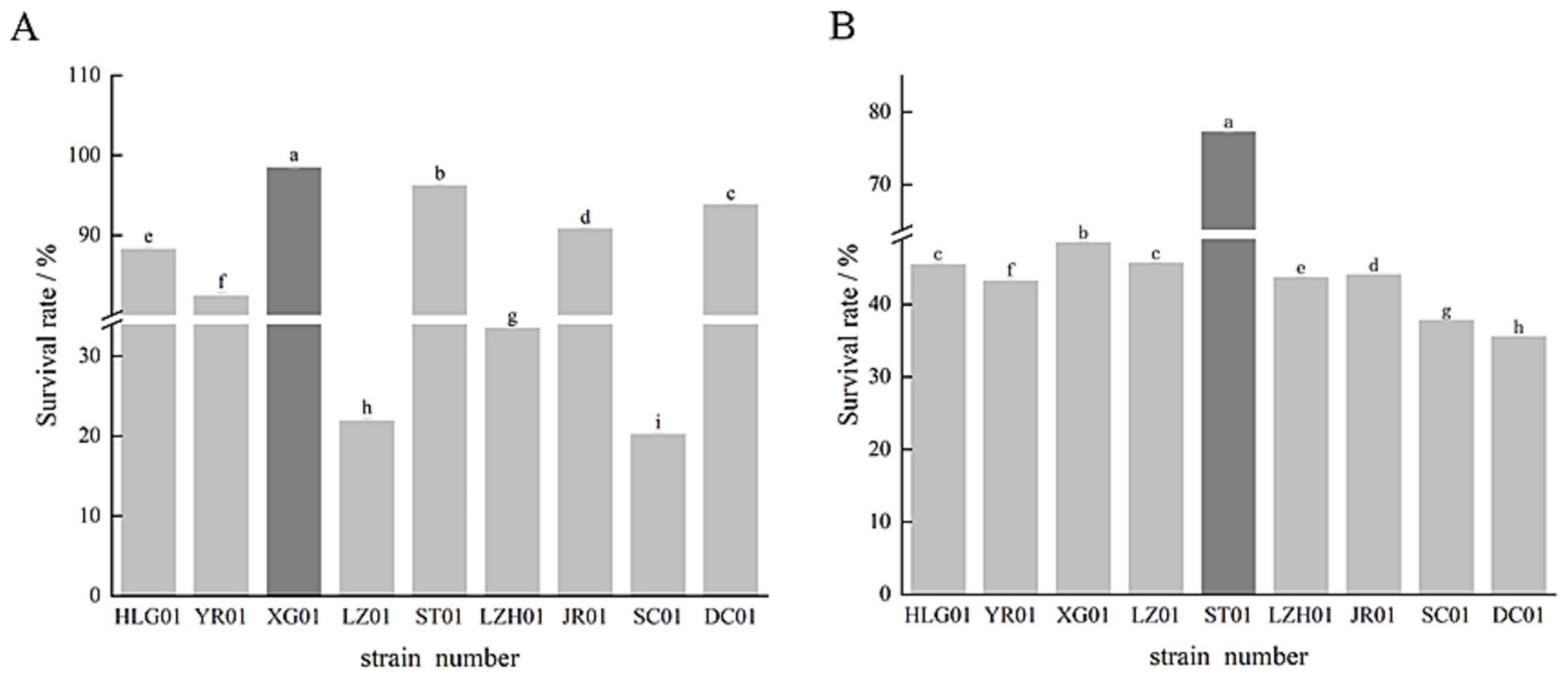
Effect of lactic acid bacteria on acid resistance survival rate **(A)**, effect of lactic acid bacteria on bile salt resistance survival rate **(B)**.

The tolerance of the screened strains to acid and bile salts is depicted in Figure 3. As shown in Figure 3A, XG01 demonstrated the strongest acid resistance with a survival rate as high as 98.5%. Except for LZ01, LZH01, and SC01, which had relatively low survival rates below 40%, the remaining seven strains all achieved survival rates exceeding 80%. From Figure 3B, it can be inferred that among the 10 lactic acid bacteria strains, ST01 exhibited the highest bile salt tolerance at 77.3%. In contrast, SC01 and DC01 showed relatively poor bile salt tolerance with survival rates of 37.8 and 35.53%, respectively. Nevertheless, eight of the Lactic acid bacteria strains maintained a bile salt tolerance survival rate above 40%. Taking into account the survival rates under both acid and bile salt stress, five Lactic acid bacteria strains, namely HLG01, YR01, XG01, ST01 and JR01, were ultimately selected for their superior acid and bile salt resistance.

### 3.3 Lactic acid bacteria colony morphology and microscopic observation, and molecular biology identification

#### 3.3.1 Observation on colony and cell morphology of lactic acid bacteria

**Figure 4:**
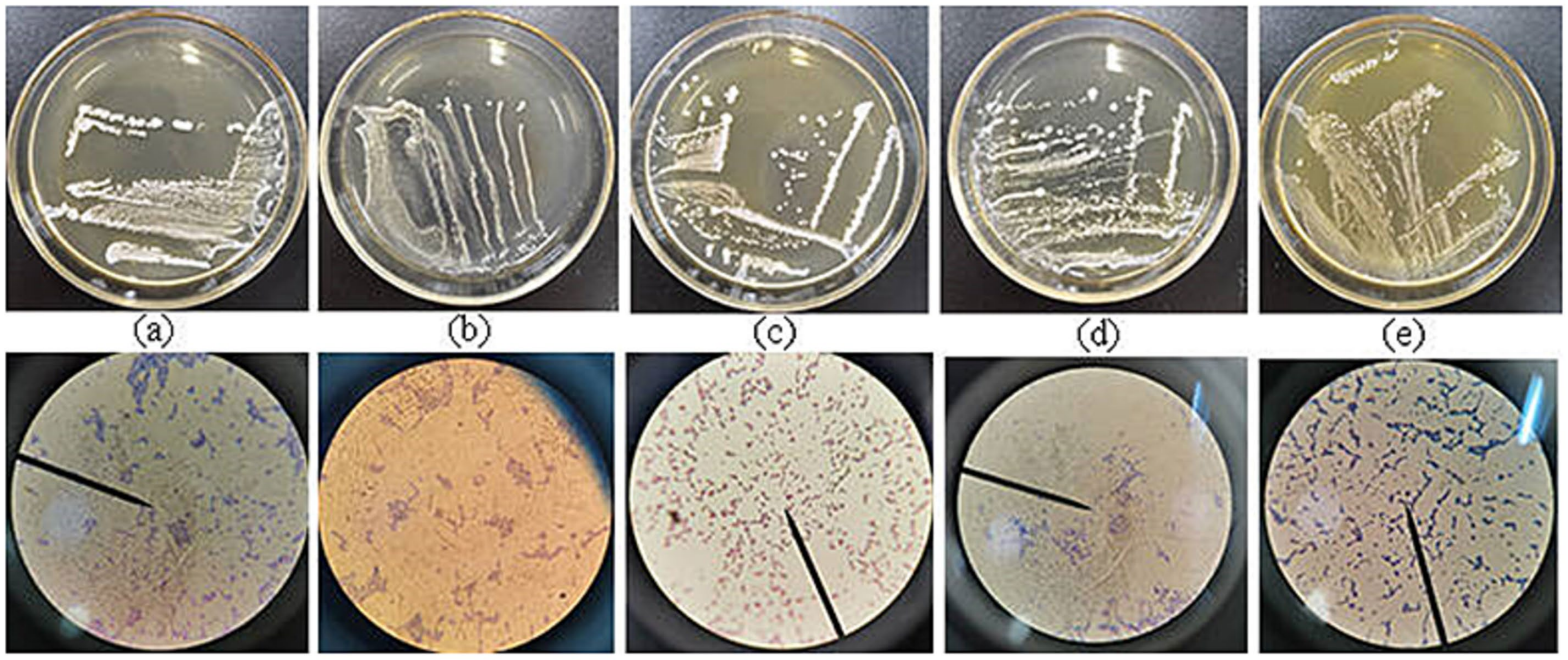
Morphological identification of colonies and cells of 5 strains of lactic acid bacteria **(a)**: HLG01; **(b)**: YR01; **(c)**: XG01; **(d)**: ST01; **(e)**: JR01.

The 5 lactic acid bacteria strains exhibited milky-white spherical colonies, smooth-surfaced and slightly elevated in the center. After Gram staining, HLG01 and ST01, strains were bacillus-like, while YR01, JR01, and XG01 strains were coccoid, as presented in Figure 4.

#### 3.3.2 Identification of lactic acid bacteria 16S rDNA gene

**Figure 5:**
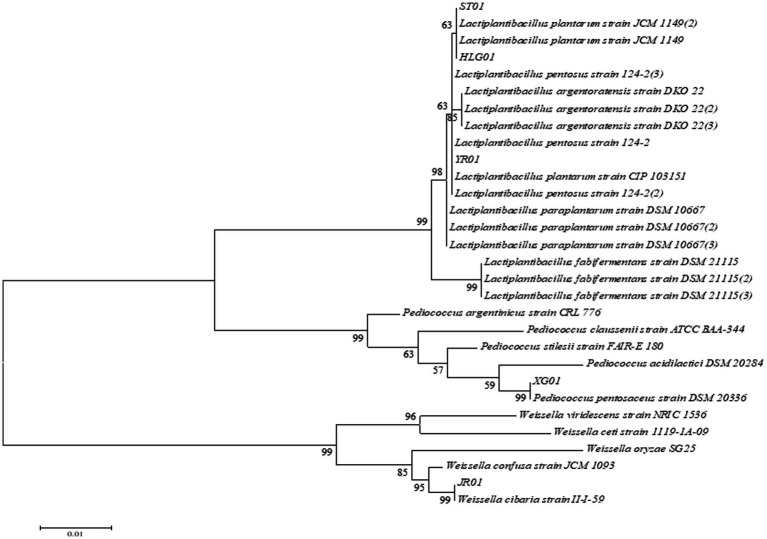
Phylogenetic tree based on 16S rDNA sequence of 5 isolates.

Five strains of lactic acid bacteria with α-glucosidase inhibitory activity and acid and bile salt resistance were selected for 16S rDNA gene sequencing. The results were compared with the NCBI gene library, and the strain with the highest homology was used as the identification result. The comparison results of the five lactic acid bacteria will be shown in Table 1. The appropriate reference strain sequence was selected, and the phylogenetic tree was constructed using MEGA 6.0 to show the genetic relationship between the strains. The phylogenetic tree is shown in Figure 5.

**Table 1:** The contrast of lactic acid bacteria identification results.

Strain number	Strain genus name	Gene pool number	Homology
HLG01	*Lactiplantibacillus plantarum*	NR115605.1	100%
YR01	*Lactiplantibacillus plantarum*	NR104573.1	99.58%
XG01	*Pediococcus pentosaceus*	NR042058.1	99.18%
ST01	*Lactiplantibacillus plantarum*	NR115605.1	99.93%
JR01	*Weissella cibaria*	NR032964.1	99.73%

As indicated in Table 1, the 16S rDNA gene sequences of strains HLG01 and ST01 display 100 and 99.93% homology with the *Lactiplantibacillus plantarum* 16S rDNA gene sequence (gene bank accession number NR115605.1), identifying them as *L. plantarum*. Strain YR01 shows 99.58% homology with the *L. plantarum* 16S rDNA sequence (NR104573.1) and is also identified a*s L. plantarum*. The 16S rDNA gene sequence of strain XG01 exhibits 99.18% homology with *Pediococcus pentosaceus* and is identified as such. Strain JR01 is identified as *Weissella cibaria*, with 99.73% homology to the *W. cibaria* 16S rDNA gene sequence (NR032964.1).

## 4 Discussion

Lactic acid bacteria are ubiquitously present in fermented foods, dairy products, and the human digestive tract. They have extensive applications in the food and medical sectors. During fermentation, these bacteria produce lactic acid, thereby lowering the pH of food and inhibiting the proliferation of harmful microorganisms. This action effectively extends the shelf life of food products. Lactic acid bacteria also offer numerous health benefits, such as modulating intestinal flora balance and preserving gut health (Zhou et al., 2022).

Moreover, certain strains of fermented juices possess hypoglycemic properties (Zhong et al., 2021). A series of studies have highlighted that specific lactic acid bacteria strains, including *Lactobacillus sake* MBEL1397 (Kwun et al., 2020), *Lactobacillus mali* K65, *Lactobacillus casei* K9, *Leuconostoc mesenteroides* K67 (Kim et al., 2021), *Lactobacillus casei* 2W, and *Lactobacillus rhamnosus* Z7 (Chen et al., 2014), demonstrate pronounced α-glucosidase inhibitory activity alongside desirable probiotic attributes. Consequently, these strains hold promise as potential probiotics for blood glucose management. The food industry is anticipated to benefit from the application of these strains.

In this study, five strains of Lactic acid bacteria with α-glucosidase inhibitory Lactic acid bacteria strains were identified. Specifically, HLG01, YR01, and ST01 were classified as *L. plantarum*, XG01 as *P. pentosaceus*, and JR01 as *W. cibaria.* Notably, extensive domestic and international research has indicated that α-glucosidase-inhibiting lactic acid bacteria predominantly belong to the species *Lactobacillus paradryus* (Dang et al., 2018), *L. plantarum*, and *Enterococcus faecalis* (Amirsaidova et al., 2021). This study demonstrates, for the first time, that *W. cibaria* exhibits α-glucosidase inhibitory activity, which has not been reported in other related studies.

In this study, it was found that the lactic acid bacteria cell extracts of HLG01, YR01, ST01 and XG01 could better inhibit the activity of α-glucosidase, and the inhibition rates were 39.27, 38.6, 38.53, and 34.4%, respectively. The lactic acid bacteria fermentation supernatant of JR01 could better inhibit the activity of α-glucosidase, and the inhibition rate reached 33.8%. And there are differences in inhibitory activity between the strains. These differences in inhibitory activity may be attributed to the different binding sites of inhibitors caused by different enzyme structures from different sources (Zeng et al., 2020). Most of the α-glucosidase inhibitors are sugars or derivatives of sugars. By competitively inhibiting the activity of α-glucosidase, the rate of polysaccharide decomposition into glucose is reduced, and the absorption of sugar is slowed down accordingly, which plays a role in reducing blood sugar.

There is no clear result on the study of α-glucosidase inhibitory active components of lactic acid bacteria. According to the previous literature (Ramchandran and Shah, 2009), yoghurt fermented with exopolysaccharide-producing strains has stronger α-glucosidase inhibitory activity, and the content of exopolysaccharide is positively correlated with its hypoglycemic activity. It is speculated that this inhibitory activity may be caused by extracellular polysaccharides produced by strains. However, there is no further purification of exopolysaccharides for verification. Therefore, the active components, inhibition mechanism and influencing factors of enzyme inhibitors in the supernatant of lactic acid bacteria need further study. The fermentation supernatant primarily consisted of MRS broth medium, which includes polysaccharides, lipids, carbon sources, organic acids, and additional components. According to Sreepathi et al. (2023), hydroxycitric acid was identified as a key α-glucosidase inhibitor. Previous screening studies of LAB strains producing hydroxycitric acid revealed that this metabolite exhibits potent α-glucosidase inhibitory activity during fermentation. The observed variation in α-glucosidase inhibition by the fermentation supernatant may arise from interactions between medium components and LAB-derived metabolites.

Significant variations in α-glucosidase inhibition rates were observed among fermentation supernatants from different LAB strains. Wang X. et al. (2021) and Wang Y. et al. (2021) thought that the inhibition rate of different strains was significantly different in the study of α-glucosidase inhibition rate, which may be the reason for the inhibition of α-glucosidase by extracellular polysaccharide and lactic acid produced by Lactic acid bacteria.

To exert their beneficial effects, LAB must first survive passage through the acidic gastric environment and resist bile salt degradation before reaching the intestinal tract. Certain LAB strains demonstrate remarkable acid tolerance, enabling them to not only withstand low pH conditions but also actively synthesize beneficial metabolites, including lactic acid and various bioactive compounds.

These microbial-derived compounds contribute to maintaining gut microbiota homeostasis and enhance immune modulation (Yan and Polk, 2020). Dysbiosis of the gut microbiota has been strongly associated with both the pathogenesis and progression of diabetes mellitus. Through microbiota modulation, LAB may promote intestinal health and attenuate inflammatory responses, thereby potentially offering therapeutic benefits for diabetes management.

Under normal physiological conditions, the pH value of human gastric juice is usually 1.3 ~ 1.8. After eating, the gastric juice is diluted by food, and its pH value can rise to 3.5. In general, the residence time of food in the stomach is about 1 ~ 2 h. Gastric acid plays an important defensive role in the internal environment of the stomach, which can inhibit or kill the microorganisms entering the stomach with food. Usually, the higher the acidity of gastric juice, the more significant the inhibitory effect on microorganisms. In this experiment, the survival rate of 5 strains of lactic acid bacteria was higher in the simulated gastric acid environment, and the survival rate was more than 80% at pH 2.5, which was consistent with the basic characteristics of probiotics. This finding indicates that these lactic acid bacteria strains have good acid resistance and can maintain a high survival rate in the gastric acid environment, which has the potential to be used as probiotics in related fields.

Mulaw et al. (2019) screened 4 strains of lactic acid bacteria with pH 2.5 tolerance from 56 strains of probiotics, and the lowest survival rate was 77% and the highest was 91%, which was not much different from the 5 strains with a higher survival rate in this study. It can be seen that the acid resistance of the 5 strains in this experiment is good, which may be related to the metabolic pathways involved in various biological enzymes in the bacteria (Wang X. et al., 2021; Wang Y. et al., 2021).

Bile salt is a sodium salt or potassium salt formed by the combination of bile acid secreted by hepatocytes and glycine or taurine. In the human body, bile mainly contains 75% sodium glycocholate and 25% sodium taurocholate. The concentration of bile salts in the small intestine is usually 0.03% ~ 0.3%. This high concentration of bile salts can produce high osmotic pressure outside the cell, thus affecting the bacterial cells (Xie et al., 2019). In this experiment, the bile salt tolerance characteristics of 5 strains of lactic acid bacteria were evaluated. The results showed that the survival rate of these lactic acid bacteria was more than 40.0% under the simulated pig bile salt concentration of 0.3%, and the highest survival rate reached 77.3%. The results showed that these lactic acid bacteria strains had good bile salt tolerance, which was consistent with the basic characteristics of probiotics. This bile salt tolerance is essential for the survival and colonization of probiotics in the intestine, because bile salts have an inhibitory effect on microorganisms in the intestine. Therefore, these bile salt-tolerant lactic acid bacteria strains have potential value in probiotic applications.

Angelescu et al. (2019) have good resistance to high concentrations of bile salts (up to 2%). The percentage of living cells is about 50% or more, which is different from the survival rate of each strain in this study, which may be related to the composition of fatty acids in lactic acid bacteria (Bustos et al., 2018). However, although *in vitro* experiments can evaluate the acid and bile salt tolerance of probiotics, these experimental results are not sufficient to fully predict the actual function of probiotics in the human body. At present, one of the main limitations of this experimental probiotic study is the lack of *in vivo* validation. Therefore, the specific mechanism remains to be further studied. In this study, probiotic lactic acid bacteria with α-glucosidase inhibitory activity, good acid and bile salt resistance, and hypoglycemic potential were isolated and screened from traditional fermented foods from different sources in Guizhou, which provided a basis for the subsequent development of related functional foods or probiotic health products.

## 5 Conclusion

In this study, 27 strains of lactic acid bacteria were isolated from traditional fermented foods in Guizhou, and the α-glucosidase inhibitory activity of their fermentation supernatant and cell disruption extracts was screened. The results showed that 10 strains had α-glucosidase inhibitory activity, and 5 strains showed good acid and bile salt tolerance. The acid resistance of the XG01 strain was the best, and the survival rate was as high as 98.5%. ST01 strain had the strongest bile salt tolerance, which was 77.3%. In terms of α-glucosidase inhibitory activity, the inhibition rates of lactic acid bacteria cell extracts of *L. plantarum* (HLG01, YR01, ST01) and *P. pentosaceus* XG01 were 39.27, 38.6, 38.53, and 34.4%, respectively, while the inhibition rate of lactic acid bacteria fermentation supernatant of *W. cibaria* JR01 reached 33.8%. Among them, *W. cibaria* JR01 is the first lactic acid bacteria that has been reported to inhibit α-glucosidase activity. The results of this study showed that the metabolites of 5 strains of lactic acid bacteria with good tolerance had potential hypoglycemic function. Subsequent experiments will screen out effective active ingredients from lactic acid bacteria fermentation supernatant and lactic acid bacteria cell extract, and isolate and purify them to determine their chemical structure. Furthermore, we further studied its effects on glucose tolerance and postprandial blood glucose fluctuation *in vivo* through animal models, to provide theoretical support for the development of new hypoglycemic drugs or functional foods.

## Data Availability

The original contributions presented in the study are included in the article/supplementary material, further inquiries can be directed to the corresponding author.
